# Innovative diagnostic device for thickness measurement of conveyor belts in horizontal transport

**DOI:** 10.1038/s41598-022-11148-1

**Published:** 2022-05-04

**Authors:** Agata Kirjanów-Błażej, Ryszard Błażej, Leszek Jurdziak, Tomasz Kozłowski, Aleksandra Rzeszowska

**Affiliations:** 1 Faculty of Computer Science and Telecommunications, Wroclaw University of Science and Technology, Wrocław, Poland; 2 Faculty of Mining Geoengineering and Geology, Wroclaw University of Science and Technology, Wrocław, Poland

**Keywords:** Engineering, Electrical and electronic engineering, Mechanical engineering

## Abstract

Diagnostics of conveyor belts used in horizontal transport without the need to take the belt off the conveyor and test it in laboratory conditions is an important aspect in mining plants (Jurdziak et al., Adv Intell Syst Comput, 835:645–654, 2019). Current testing, and thus obtaining knowledge about the current thickness of the conveyor belt covers, allows for control accelerated changes. It also avoids emergency stoppages in the operation of the conveyor and enables economically justified planning of a break in its operation. The article presents the concept of the first in Poland mobile device for measuring the thickness of conveyor belts in motion, implemented as part of the NCBR project (No. 0227 / L-10/2018 [LIDER program, Transport Przemysłowy i Maszyny Robocze, 1(47)/2020, pp. 60–61]), and also presents the measurement results obtained thanks to the laboratory version of the device.

## Introduction

**Figure 1:**
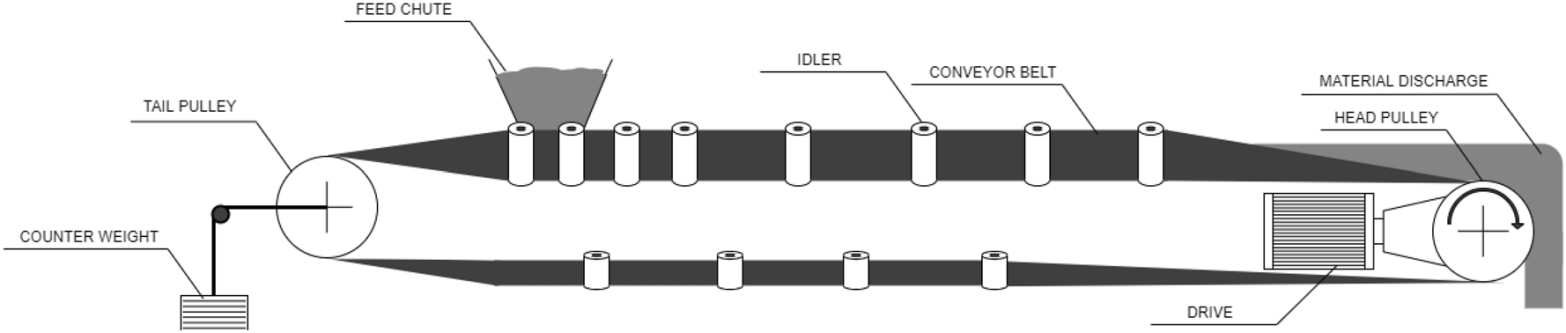
Diagram of the conveyor belt.

The lifetime of the conveyor belt depends on many factors presented in the literature^3^—including the type of material transported, the specificity of the transport point as well as the length and condition of the conveyor belt. Figure 1 shows a construction diagram of a belt conveyor used in mining^4^.

The most expensive and emergency part of the conveyor is the belt. It is estimated that its cost is about 60% of the cost of the entire conveyor^5^. During operation, it is exposed to unfavourable phenomena that significantly reduce its durability—abrasion, stretching, tearing, cutting, cracking, delamination and uneven working conditions due to variable operating conditions (temperature, atmosphere, load, UV rays, belt tensioning force, friction). The requirements for manufacturers of conveyor belts define the belt as a high-quality product, which in turn translates into its cost. The more important is its diagnosis and quick detection of possible irregularities when it is still possible to remove them. A potential failure of a belt conveyor generates costs related not only to its repair, but also related to forced stoppage in transport and production losses^5,6^.

The specificity of NDT (non-destructive testing) assumes that during the examination of the object (here the conveyor belt) it does not degrade, and its structure and properties do not change. NDT-based methods are gaining more and more attention in diagnostic of the technical condition of belt conveyors^7^. Previously, the research focused only on the individual components of the conveyor: belts^8–17^, drives^18^, idlers^19^ or gearboxes^20,21^.

Many researchers around the world have developed many systems for the diagnosis of conveyor belts^5^. Some of the available methods are used to diagnose the condition of covers, others to detect damage to the steel core inside the rubber^22,23^. In the era of Industry 4.0, installing a sensor on the tested object, and then collecting data and then processing them, leads to the improvement of the research process and control of the continuity of the work point and various types of threats^1^.

The aim of the project implemented at the Wroclaw University of Science and Technology is to develop a new device for measuring thickness and assessing changes in the cross and longitudinal profile of conveyor belts, as well as to create its industrial version.

## The idea of operation of the device

The most important part of the designed device is the ultrasonic distance sensor. The sensor consists of two piezoelectric elements, one in transmitter mode and the other in receiver mode. The transmitter emits an ultrasonic wave, i.e. a wave with a frequency above the upper limit of audibility for the human ear (above 20 kHz), which propagates through space and reflects off the obstacle. The echo is picked up by the receiver, and the time from transmitting the wave to receiving it, measured inside the sensor, clearly determines the distance between the sensor and the obstacle. The emitted ultrasonic wave propagates through the air at a constant and known speed, depending on the parameters of the medium—mainly temperature, but also humidity. The sound wave is a mechanical wave, so it propagates as a disturbance of the medium. Temperature is defined as the average kinetic energy of a molecule, so a change in temperature changes the speed of the molecules and thus the speed of wave propagation. In order to compensate for the variable speed of the ultrasonic wave depending on the temperature, the ultrasonic sensors have a built-in temperature compensation functionality^24^.

Industrial ultrasonic sensors available on the market use frequencies from 25 to 500 kHz, and the sensor's operating frequency is inversely proportional to the distance range^25^—waves with lower frequencies are used to detect objects at greater distances, and waves with higher frequencies are used to detect objects closer.

**Figure 2:**
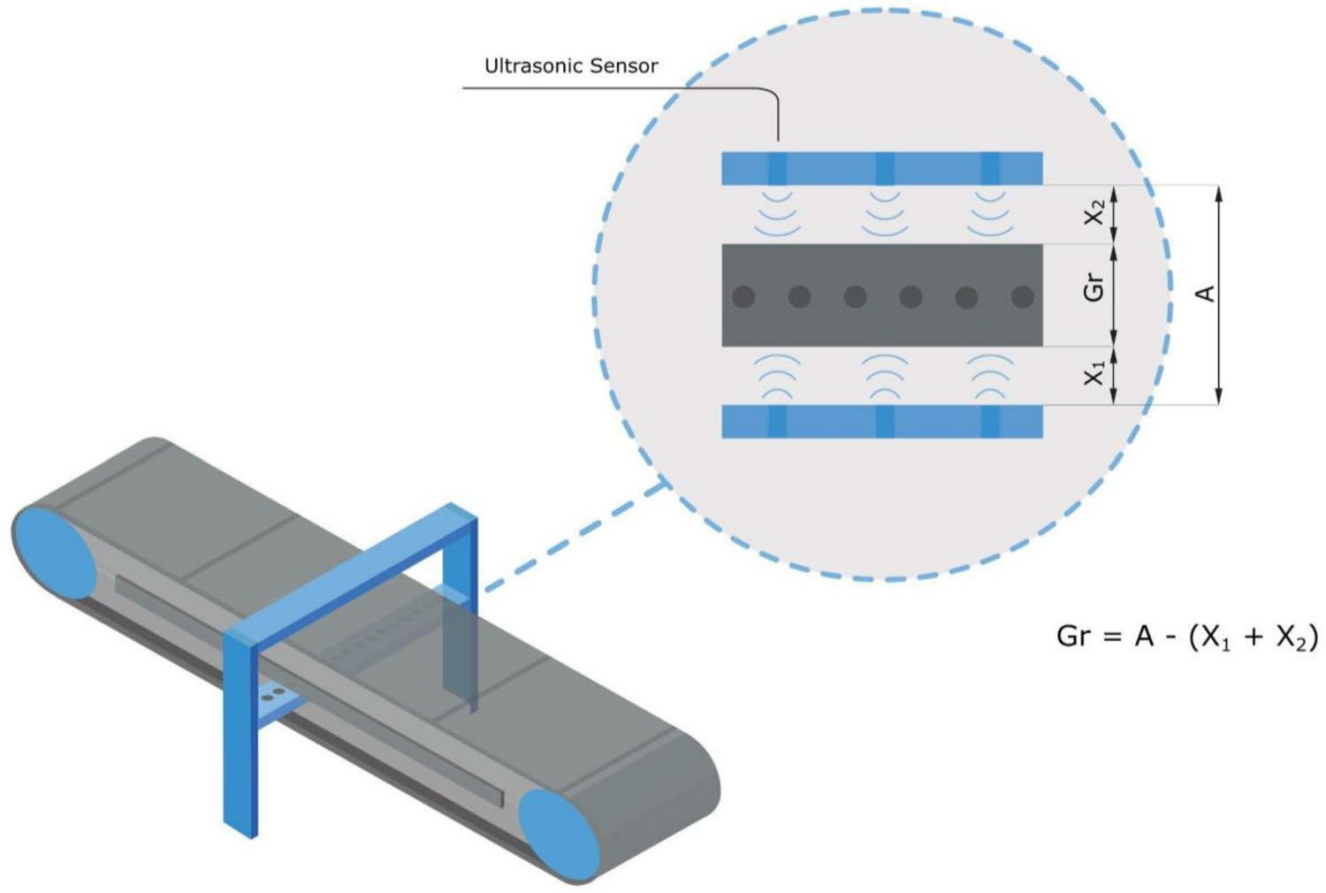
The idea of differential measurement^28^.

The idea of thickness measurement is based on a differential measurement—an ultrasonic sensor is placed both above and below the tested object. The idea of the measurement is presented in Fig. 2. The sensor placed above the tested object determines the distance described by the symbol $X_{1}$, and the sensor placed under the object—$X_{2}$. Knowing the distance between the sensors above and below the object (here marked with the letter $A$) clearly determines the thickness of the object at a given location^6,26,27^.

**Figure 3:**
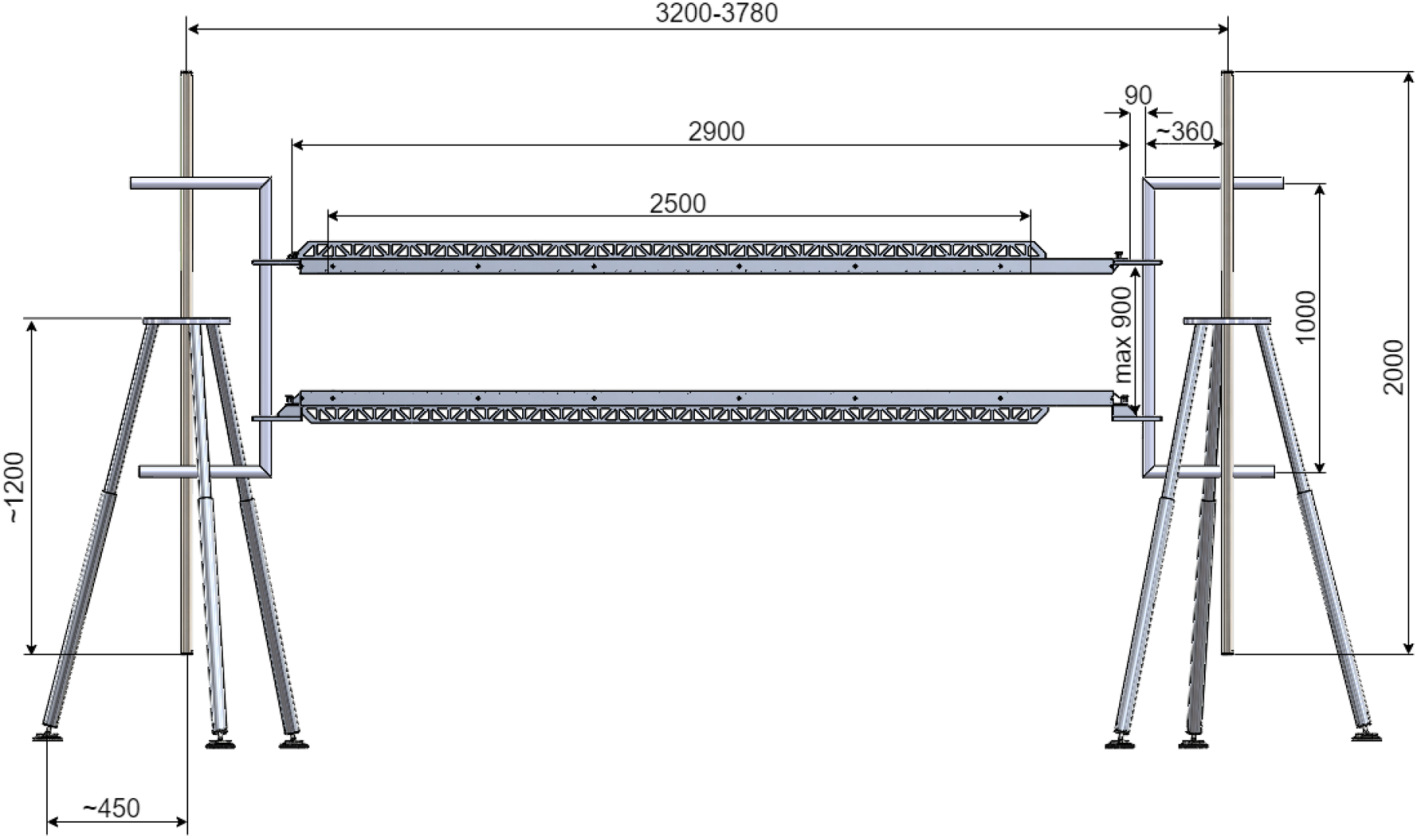
Diagram of the designed device.

To ensure the operation of the system, ultrasonic sensors are placed on two measuring heads, between which the conveyor belt moves. The sensors are placed at equal distances from each other, along a length of 2.5 m, with 50 sensors on each of the measuring heads. The heads are attached to external tripods, the position of which can be adjusted by changing the spacing of the tripod legs. This method of mounting the measuring heads allows the measurement results to be made independent of vibrations, belt impacts that may occur during the tests, as well as deformations of the conveyor structure occurring over time, which may hinder or prevent effective mounting of the device. Installation of the designed system on its own structure allows the system to be installed in any flat section of the belt, and thus makes its operation independent of the conveyor's supporting structure^29^. A diagram of the designed system with dimensions is shown in Fig. 3.

## Device prototype

**Figure 4:**
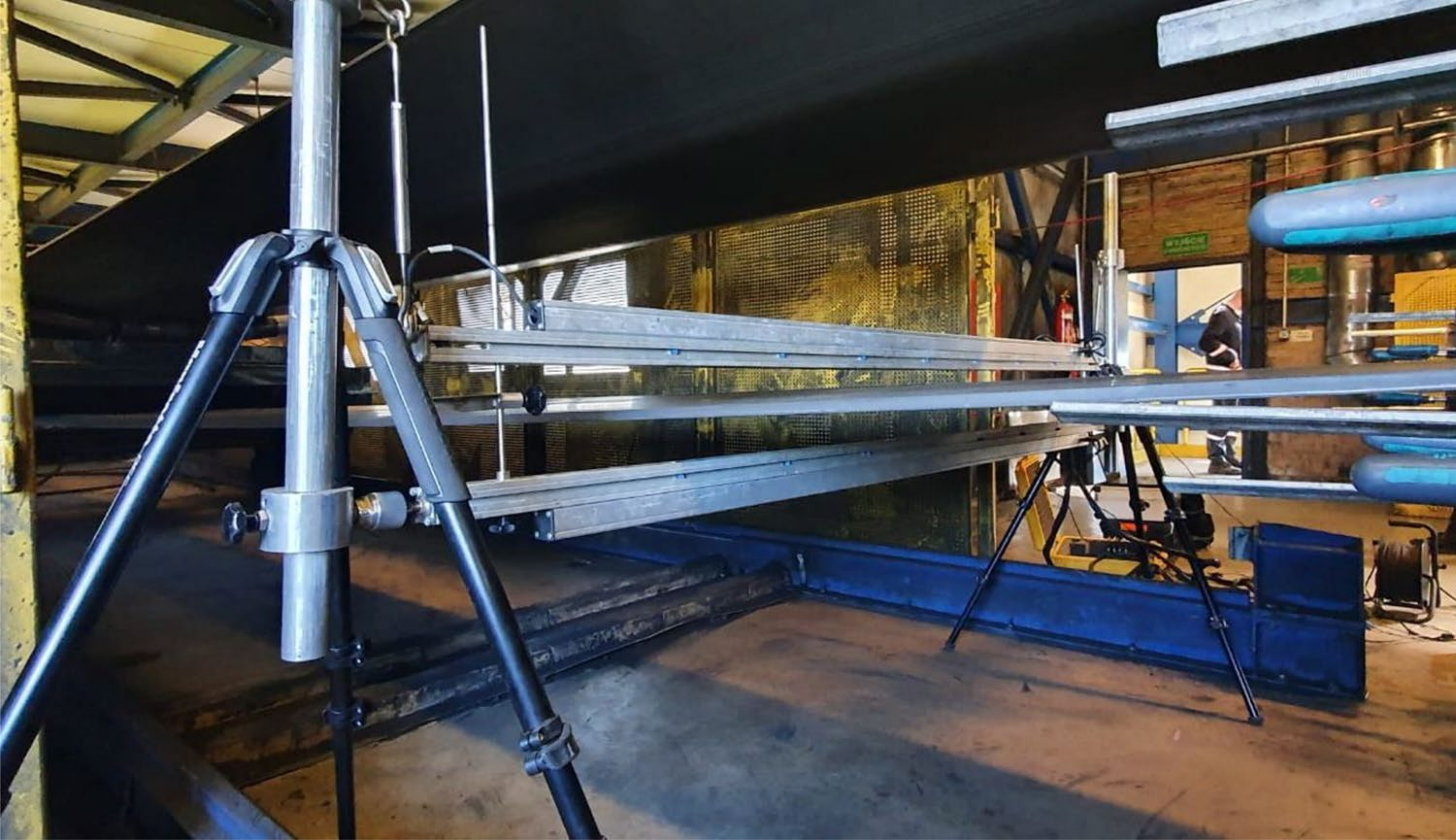
The prototype installed on a conveyor belt in the mine during measurements.

In order to check whether the proposed idea of measuring the thickness of the conveyor belt with the use of ultrasonic sensors will meet the challenges it faces, a laboratory version of the device was made (Polish patent, No. 228973. *Device for measuring the thickness and assessing changes in the cross and longitudinal profile of the conveyor belt*). The purpose of building a prototype version was to test the measurement method and to select the components of the industrial device^29^. The prototype was tested both in laboratory conditions, at the Belt Transport Laboratory of the Wroclaw University of Science and Technology, and in real conditions—in the mine. The measuring heads of the prototype system include 7 pairs of ultrasonic sensors arranged on two measurement heads at a distance of 250 mm from each other, over a span of 1.5 m. The prototype device additionally uses two laser distance sensors at the beginning and end of the upper measuring head. The task of the laser rangefinders used was to help maintain the parallelism of the measuring heads—the heads are located parallel to each other when the indications of both laser sensors are the same. Figure 4 shows the prototype version during measurements in the mine.

Another component of the prototype system is the application that receives raw measurement data. After processing the data, the application generates a report on the technical condition of the belt, which includes graphs specifying the longitudinal and cross profile in a place selected by the user, a three-dimensional image of the tape, as well as characteristic parameters for the selected profile—average thickness of the belt, maximum and minimum thickness. The measurement results form a data grid, but due to the few placement of sensors along the measuring head, when generating a three-dimensional image and a contour map, the grid was densified by interpolating the values between the cubic method ("Cubic Hermite spline"), according to which, the value at the query point is based on the cubic interpolation of values at adjacent grid points in each appropriate dimension. Higher density of sensors allows for a denser measurement grid, and thus a better representation of the object's surface without the need to use cubic interpolation between its nodes. Then it is enough to use only linear interpolation to calculate the values between the nodes of the measurement grid.

The tests carried out on a real object in mining conditions allowed to test the prototype device, its mode of operation and software in difficult conditions—in increased humidity and dustiness. Assessing the thickness of the tape requires the installation of the device in the flat section of the belt run, and making a few full turns of the belt loop to minimize any possible measurement errors. Measurement data are saved to a file after decoding the voltage value read from the sensor output at a distance according to the specified by the manufacturer formula^30^, defined by the formula (1).

1 $$ d = a \cdot U + b $$

where $d$—sensor distance from the obstacle (mm), $U$—voltage on the sensor [V].

**Figure 5:**
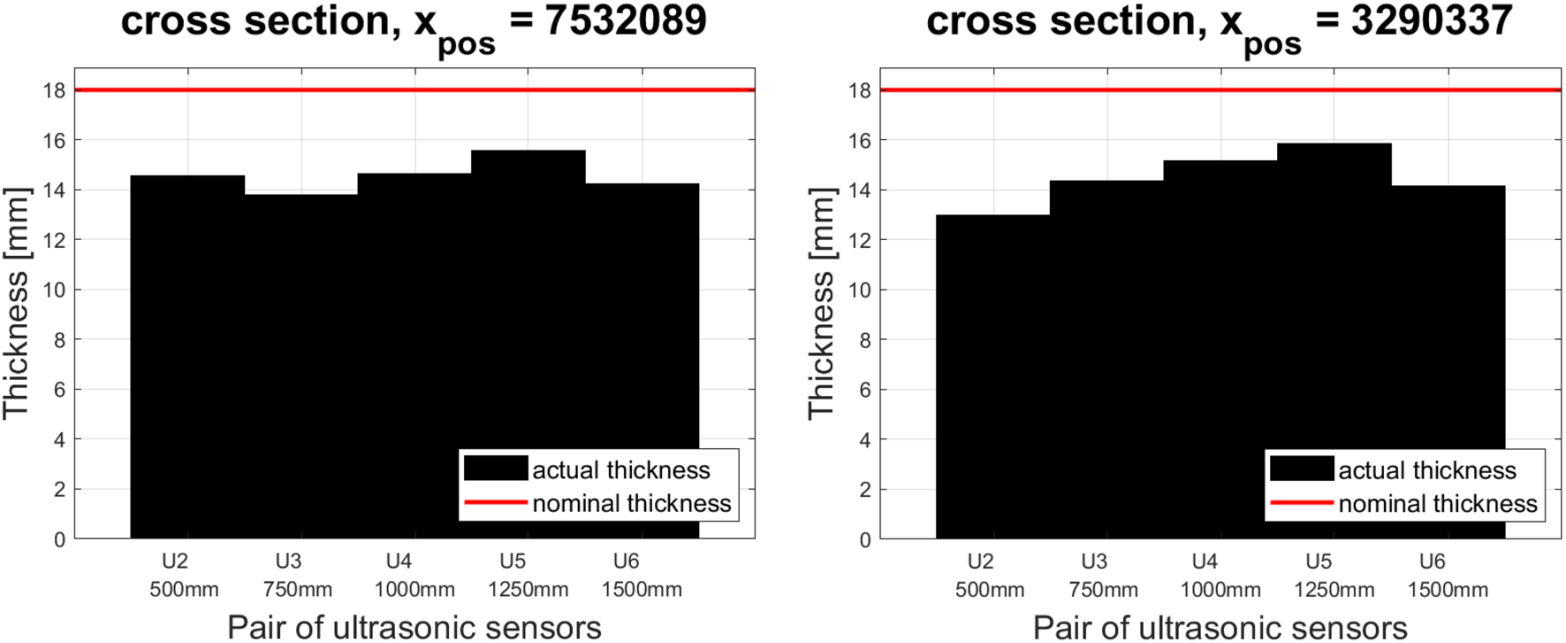
The cross-section of the conveyor belt—device prototype testing.

The analysis of measurement data allows to generate a cross or longitudinal section of the belt, as well as to plot its characteristic parameters. During the testing of the prototype, a 10-year-old conveyor belt with a nominal thickness of 18 mm was tested. The belt is used in one of the underground mines in Poland and consists of 13 sections. Figure 5 shows the cross-section of the belt at two randomly selected points along the length of the belt. The value corresponding to the nominal thickness of the belt is marked in red. Due to the fact that 7 pairs of sensors were distributed along the measuring head, but only 5 were on the width of the belt (one pair of sensors was completely outside the range (U1), and the other was on the edge of the belt (U7)), the indications of the first and the last pair of sensors were excluded from further analysis.

Table 1 presents the most important parameters for the cross-sections shown in Fig. 5.

**Table 1:** Parameters for the cross-section—testing the device prototype.

Value	x_pos = 7,532,089	x_pos = 3,290,337
Mean (mm)	14.5513	14.4969
Minimal (mm)	13.7893	12.9683
Maximal (mm)	15.5595	15.8575
Nominal (mm)	18.0000	18.0000
Relative average thickness (%)	80.8403	80.5381
Surface loss (mm^2^)	1.4591 $\cdot 10^{4}$	1.4731 $\cdot 10^{4}$
Relative thickness (%)	81.0591	81.8375

**Figure 6:**
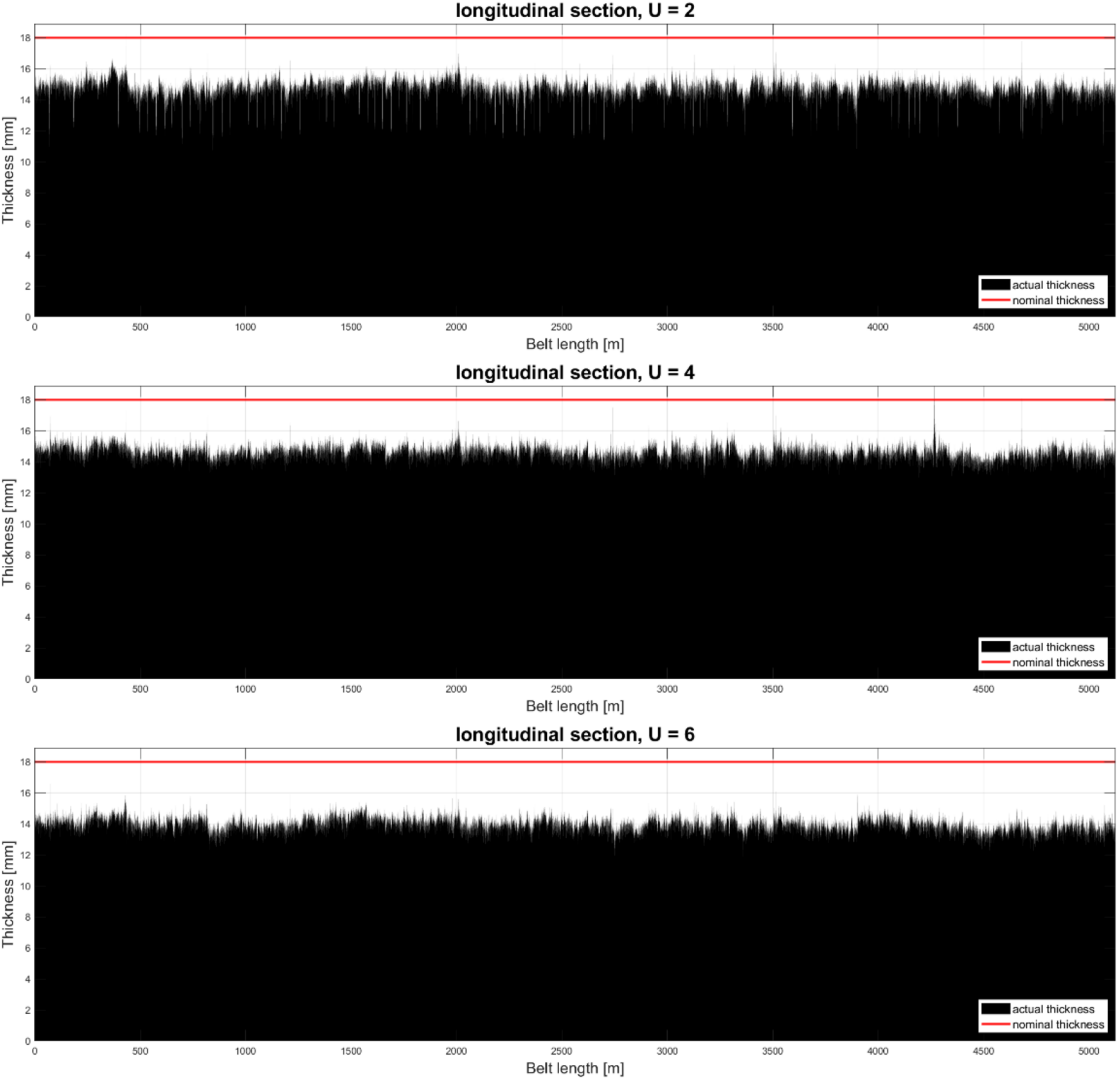
Overall longitudinal section through a conveyor belt—device prototype testing.

The loss of surface (cross or longitudinal section) was determined using the numerical integration method—the trapezoidal method. Analogous graphs and parameters can be determined for the overall longitudinal profile of the selected pair of sensors. Figure 6 shows the longitudinal sections for three pairs of sensors, and Table 2 summarizes the parameters describing each of these sections.

**Table 2:** Parameters for the longitudinal section—testing the device prototype.

Value	U2	U4	U6
Mean (mm)	14.7881	15.5067	14.0080
Minimal (mm)	10.5307	12.8820	9.3699
Maximal (mm)	18.6942	18.0548	17.1877
Nominal (mm)	18.0000	18.0000	18.0000
Relative average thickness (%)	82.1559	86.1484	77.8221
Relative wear rate [%/year]	1.7844	1.3852	2.2178

**Figure 7:**
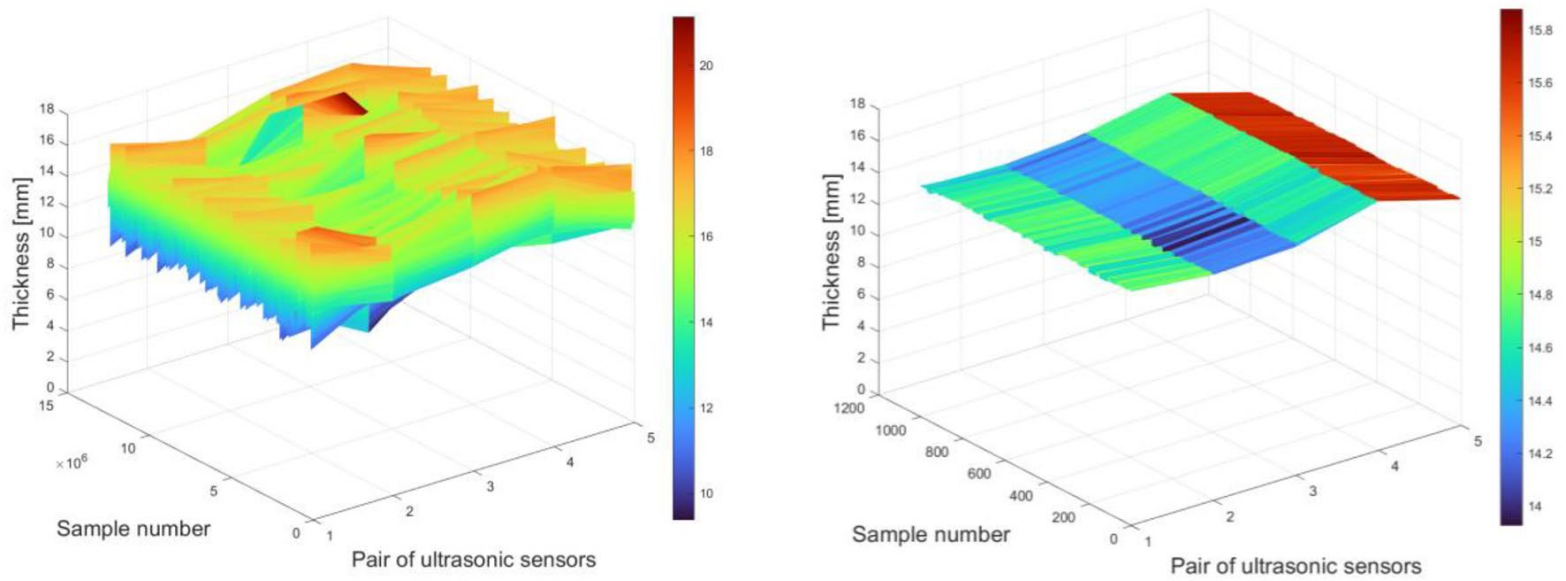
The three-dimensional image of the tape—device prototype testing.

**Figure 8:**
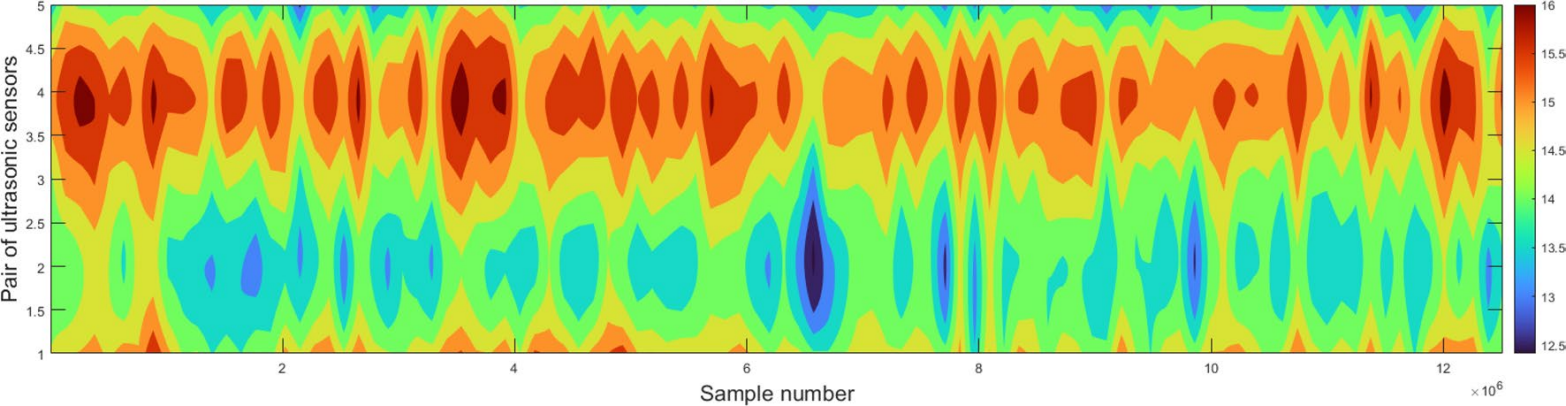
Contour map of the belt—device prototype testing.

Visualization of all measurements on a three-dimensional graph allows you to view a three-dimensional image of the tape and calculate important parameters. Figure 7 shows a three-dimensional image of the tested conveyor belt along its entire length and an approximation over a distance of 1000 samples. The measurement grid in the visualization of the three-dimensional image has not been modified and contains only nodes with values obtained in the measurements. In the case of the contour map, the size of the grid was changed by increasing it 10 times along the width of the belt and reducing it to 200 points along the length, the values were interpolated using the cubic method. The contour map is shown in Fig. 8.

**Figure 9:**
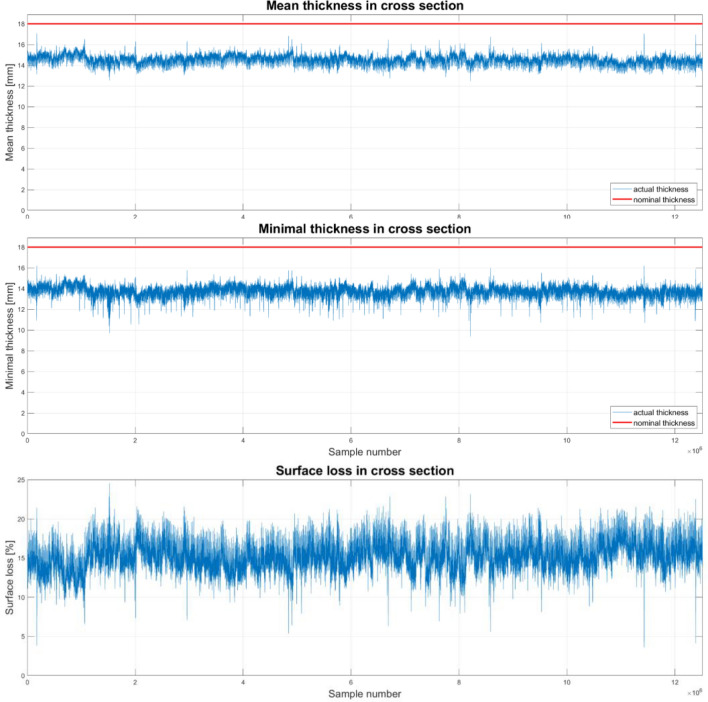
Parameters of the cross-section along the length of the belt—device prototype testing.

The parameters for the cross-section in the user-selected location for a single measurement do not support the assessment of the technical condition, but the graph showing the average value and the minimum value, as well as the percentage of surface loss along the belt, makes it easier to assess the technical condition of the belt. Such charts were determined for the tested object, and Fig. 9 and Table 3 shows their appearance.

**Table 3:** Parameters for a three-dimensional image—device prototype testing.

Value	All along	On the length segment
Mean (mm)	14.5883	14.6887
Minimal (mm)	9.3699	13.9268
Maximal (mm)	21.1373	15.8787
Nominal (mm)	18.0000	18.0000
Relative average thickness (%)	81.0463	81.6040

## Industrial version

Compared to the prototype version, the industrial version includes several modifications. Ultrasonic sensors have been changed. An incremental encoder has also been added to control the belt speed and evenly distribute the measuring grid along the tape. The method of attaching measuring heads to external stands has been improved so that the installation of the device is convenient and quick. The hardware method used in the laboratory version of the device to support the maintenance of parallelism by installing two additional laser sensors has been replaced with a software solution—before starting the measurements, all sensors are automatically calibrated. The measuring heads are spread over a given distance using model plates placed perpendicularly on both sides of the heads. The distance read by all sensors used should be the same as the width of the gauge block used. However, if the reading of any sensor pair is different from the expected value, the pair of these sensors is recalibrated automatically.

The ultrasonic sensors used in the industrial version allow for their correct operation in the range from 20 to 250 mm, and the results are saved with a resolution of 0.1 mm. The sensors can work in a wide temperature range—from − 25 to + 70 °C. The sensors have the Ingress Protection Class IP67, thanks to which they are completely resistant to dust and short-term immersion in water up to a depth of 1 m^30^.

To locate the read values along the conveyor belt, an incremental encoder was used, the operating parameters of which, combined with the operating parameters of the sensors used, allow the entire system to operate at a frequency of 100 Hz^30,31^. This frequency of operation allows to obtain a measurement with a longitudinal resolution every 1 cm for a belt running at a speed of 1 m/s and every 7 cm at 7 m/s.

**Figure 10:**
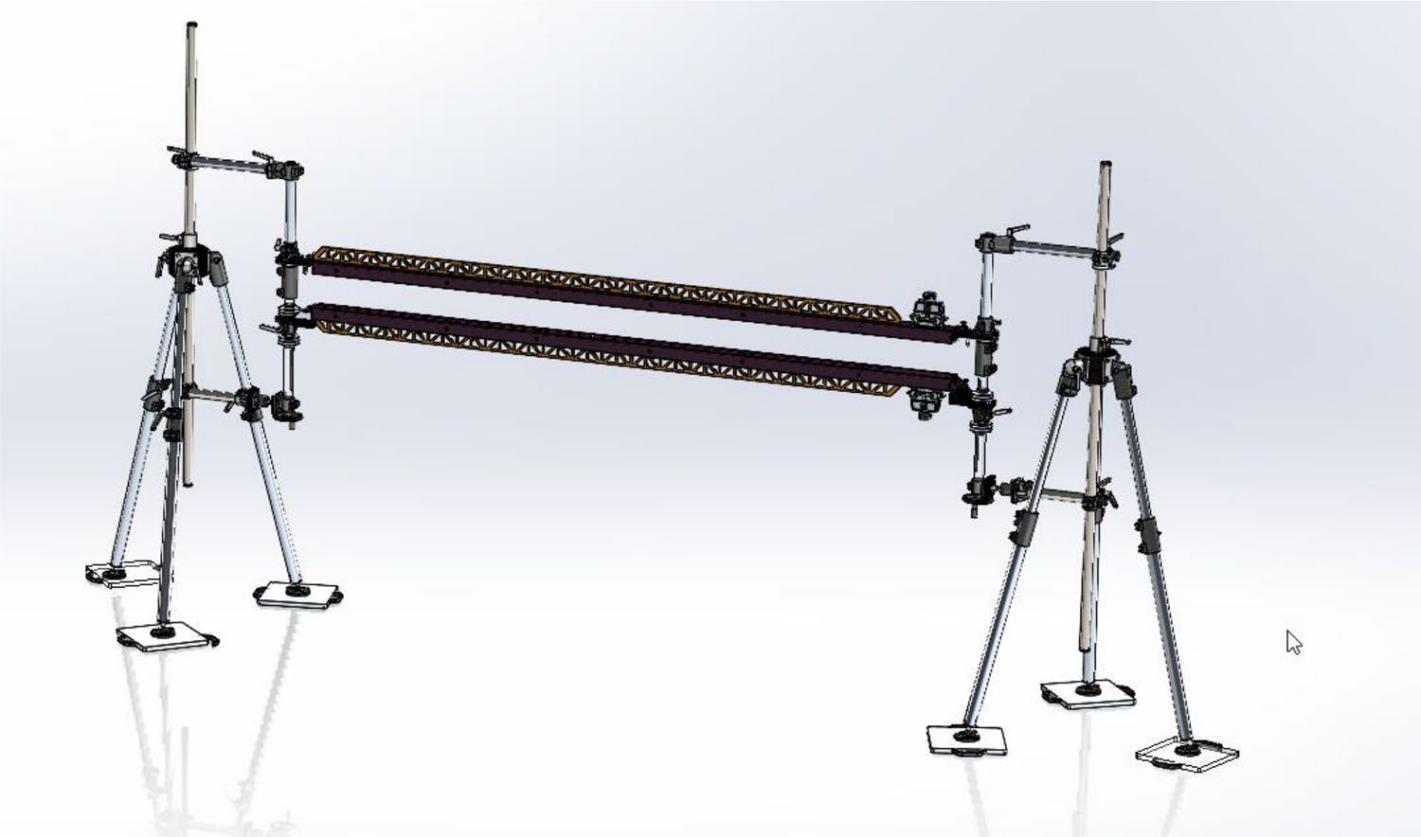
Scheme of the industrial version of the device.

**Figure 11:**
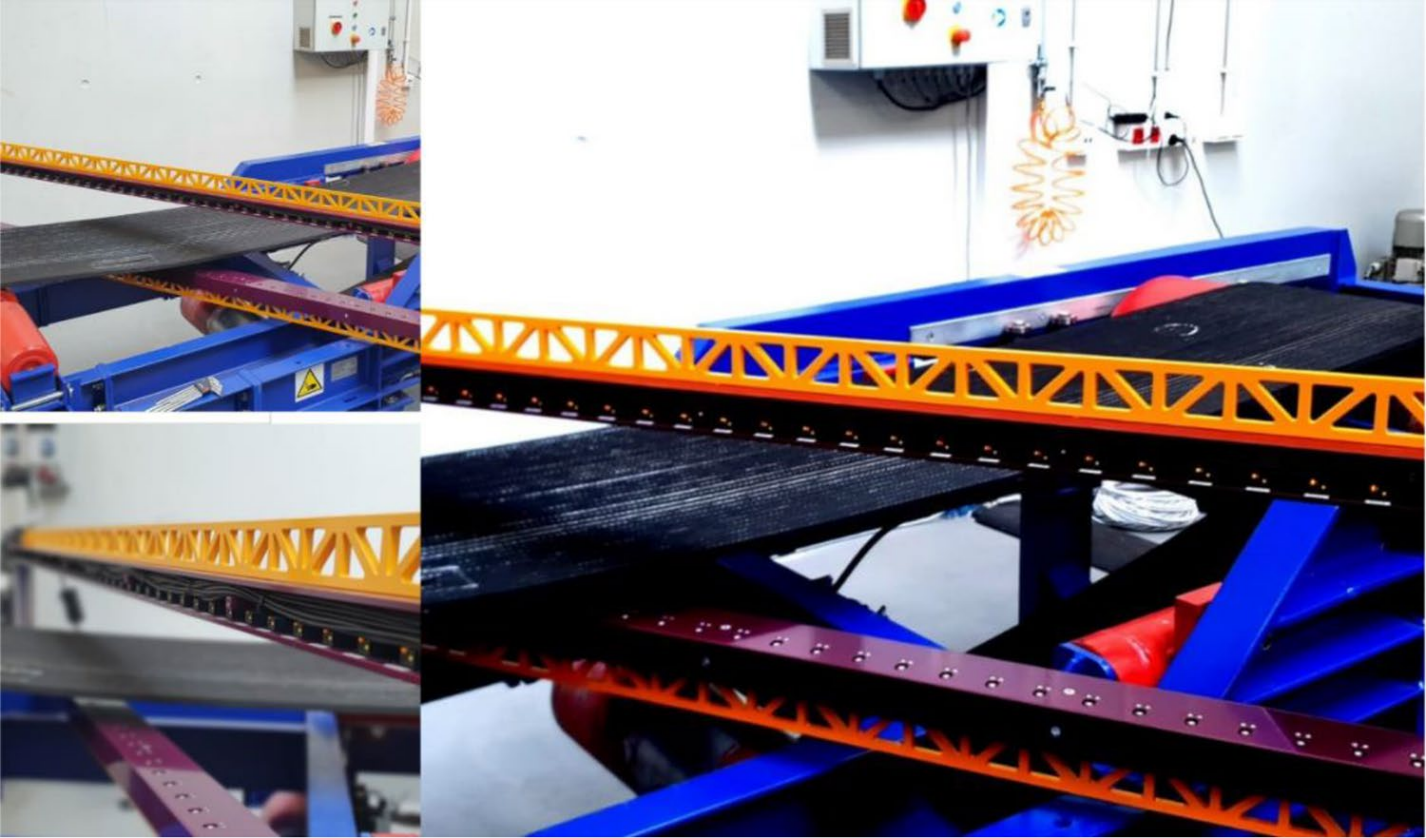
Measurements on the test conveyor.

Figure 10 shows the design of the industrial version of the device, and Fig. 11 shows the assembly of this device in a laboratory setting.

The industrial version of the device for measuring the thickness of the conveyor belt in motion is powered by a standard alternating voltage of 230 VAC, but in the event of a temporary power outage or in the absence of electricity, it can work up to 3 h thanks to the internal battery.

The industrial version of the device, like the laboratory version, includes an application that processes source data. The version of the industrial application has also been enriched in relation to the prototype application. The data received from the sensors are presented to the user in real time, although it is also possible to view the results after taking the measurements. The program, as in the case of the prototype, displays a number of graphs (longitudinal and cross profile in a place selected by the user, 3D image) and various statistics.

## Summary

Non-invasive diagnostics of conveyor belts is an important issue in maintaining transport continuity in the mining industry. The cost of belt replacement is one of the main transport costs in a mine^32^. Therefore, it is very important to control the technical condition of conveyor belts and react faster to irregularities and prevent them. It is important that, thanks to the identified and forecasting the rate of the belt wear process under specific conditions, to forecast the moment of reaching the abrasion limit and to plan downtime in mining in advance and decide to carry out preventive belt replacements based on their technical condition. As part of the project, the first in Poland mobile device for continuous measurement of belt thickness, cross and longitudinal profiles was built. Thanks to the data obtained from testing the conveyor belt, it is possible not only to determine thickness or profiles, but also to detect some damage (e.g. local abrasions and torn covers).

The advantage of the designed device is the ability to determine the thickness of the conveyor belt regardless of its type. The BeltSonic system can test both belts with a steel core and belts with a textile core, and the only limitation in the operation of the system is the need to install it in the flat section of the belt run.

The idea of the proposed differential measurement and the assembly of the measuring heads on their own design allows the system to operate independently of a number of factors that are an integral part of the operation of the belt conveyor—i.e. from vibrations, belt hits or possible deformations in the supporting structure of the conveyor.

The implementation and testing of the laboratory version allowed for its modification and, as a result, the construction of a new industrial version of the device with an improved structure. The method of attaching the heads to the conveyor structure was redesigned and an independent structure was used, which made it possible to make the system operation independent of both vibrations and the supporting structure of the conveyor on which the system will be installed.

Performing tests with the use of a prototype system and then analysis of the measurement loop allows for the determination of places most exposed to potential damage, and thus allows for faster diagnostics and removal of any irregularities. Much about the technical condition of the conveyor belt can be seen from the image of the cross-section itself (Fig. 5), because on its basis it is possible to judge at which point of the cross-section the belt abrasion rate is the highest.

The analysis of the longitudinal section through the tape allows to evaluate its profile over the entire loop, but due to the amount of measurement data spread over the length of the tape, the image may turn out to be unreadable. The longitudinal section presented in Fig. 6 is a fast-changing function, and the amount of measurement data does not allow to visually determine the place where the thickness is the smallest or largest, but it allows to assess the overall character. Determining the mean of many measurements allows to assess the changes in the average thickness along the axis, and the analysis of individual deviations allows for the local identification of damage (cover tears, punctures or abrasions) at the measurement site. Thanks to the use of an encoder, it is possible to locate these faults in the loop.

The remaining parameters listed in Tables 1 and 2 support the assessment of the technical condition, showing the user both parameters such as the minimum and maximum value, and the degree of wear of the conveyor belt. The analysis of the parameters themselves, as well as changes in these parameters over time, allows to better assess the technical condition of the tested object, as well as make decisions about a possible replacement or repair.

Due to the large amount of measurement data along the length of the conveyor belt (in the industrial version of the device there are 50 pairs of sensors), individual analysis of each cross-section is not a practical solution. Therefore, the user can determine the most important values among the selected parameters of the cross-section and the system will generate their course depending on the position of the cross-section along the length of the conveyor belt. The charts in Fig. 9 show the value of the minimum and average thickness of the belt and the percentage of surface loss. The analysis of this data will allow to identify the location of the most worn out place, and also to answer the question in which section the potential damage to the cover of the conveyor belt is located.

The industrial version of the device has been modified—ultrasonic sensors are placed at a distance of 25 mm from each other (in the laboratory version at a distance of 250 mm), which allowed for a much more accurate image of the thickness of the tape on the cross-section. The industrial version of the device has been modified—ultrasonic sensors are placed at a distance of 25 mm from each other (in the laboratory version at a distance of 250 mm), which allowed for a much more accurate image of the thickness of the tape on the cross-section.. In the next article, the results of tests of an industrial device in both laboratory and real conditions will be presented, and the results of the new tests will be interpreted and used to forecast the rate of wear and remaining belt life.
